# Supplementary material for: Green Preparation of Aminated Magnetic PMMA Microspheres via EB Irradiation and Its Highly Efficient Uptake of Ce(III)
Source: Materials (Basel). 2022 Sep 21;15(19):6553. doi: 10.3390/ma15196553 (PMC9572679; doi:10.3390/ma15196553)
Supplement: Supplementary file 1 [file materials-15-06553-s001.zip › materials-1866089-supplementary.pdf]

1. BET analysis

The N_2 adsorption-desorption isotherms of Magnetic PMMA and Magnetic PMMA-PGMA-PEI was shown in Fig.1 and the BET testing parameters were list in Table 1.The adsorption isotherm of Magnetic PMMA and Magnetic PMMA-PGMA-PEI both are type Ⅳ, and the hysteresis loop of adsorption isotherm is type H_4, which has a clear micropore filling phenomenon at low pressure,The generation of type H_4 hysteresis loop shows that a large number of mesopores appear in Magnetic PMMA and Magnetic PMMA-PGMA-PEI[1].The Brunauer-Emmett-Teller (BET) surface area, Barrett-Joyner-Halenda (BJH) pore volume and average pore diameter of Magnetic PMMAwere 38.5259 $m^2 \cdot g^{-1}$, 0.3145 $cm^3 \cdot g^{-1}$ and 3.4497 nm, respectively, according to N_2 adsorption/desorption isotherms. The BET surface area , pore volume and average pore diameter of Magnetic PMMA-PGMA-PEI were lower those of pristine Magnetic PMMA(25.2800 $m^2 \cdot g^{-1}$, 0.1843 $cm^3 \cdot g^{-1}$ and 1.7236 nm) because the grafting of organic functional groups occupied some spaces of the pores of Magnetic PMMA after chemical modification, resulting in reduced specifific surface area, pore volume and average pore diameter of Magnetic PMMA-PGMA-PEI[2].

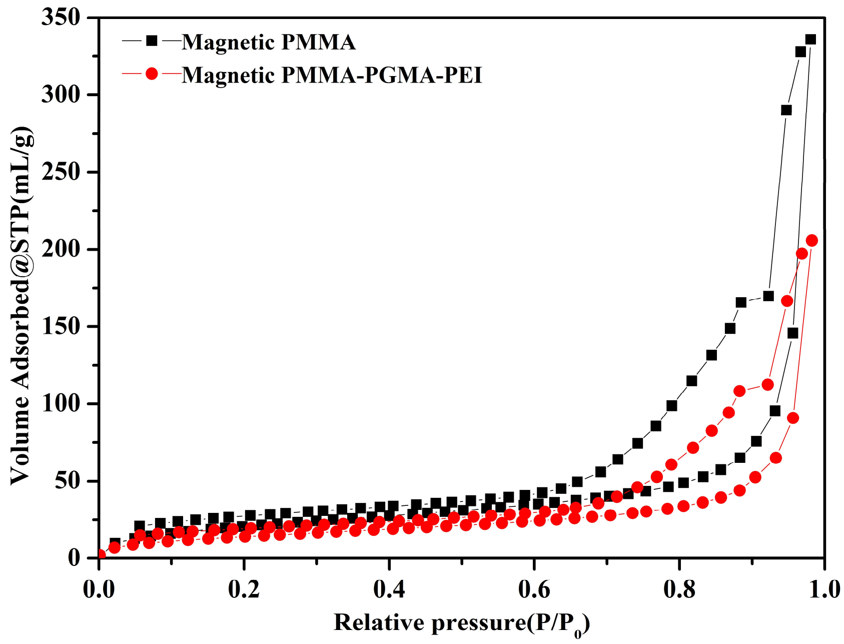

Figure S1. N_2 adsorption-desorption isotherms of Magnetic PMMA and Magnetic PMMA-PGMA-PEI

Table S1. BET testing parameters of Magnetic PMMA and Magnetic PMMA-PGMA-PEI

	Surface Area (m^2/g)	Pore Volume (mL/g)	Pore diameter Dv(d) (nm)
Magnetic PMMA	38.5259	0.3145	3.4497
Magnetic PMMA-PGMA-PEI	25.2800	0.1843	1.7236

References

[1] Ma, X.; Zhao, S.; Tian, Z.; Duan, G.; Pan, H.; Yue, Y.; Li, S.; Jian, S.; Yang, W.; Liu, K.; He, S.; Jiang, S. MOFs meet wood: reusable magnetic hydrophilic composites toward efficient water treatment with super-high dye adsorption capacity at high dye concentration. *Chem. Eng. J.* **2022**, *446*, 136851.

[2] Zhang, Y.N.; Guo, J.Z.; Wu, C.; Chen, L.; Li, B. Enhanced removal of Cr(VI) by cation functionalized bamboo hydrochar. *Bioresour. Technol.* **2022**, *347*, 126703–126709.
